# Examining Male Predominance of Severe COVID-19 Outcomes: A Systematic Review

**DOI:** 10.1089/andro.2022.0006

**Published:** 2022-07-15

**Authors:** David K. Twitchell, Michael B. Christensen, Geoffrey Hackett, Abraham Morgentaler, Farid Saad, Alexander W. Pastuszak

**Affiliations:** ^1^ University of Utah School of Medicine, Salt Lake City, Utah, USA.; ^2^ Division of Urology, Department of Surgery, University of Utah School of Medicine, Salt Lake City, Utah, USA.; ^3^ Department of Men's Health, Little Aston Hospital, Sutton Coldfield, United Kingdom.; ^4^ Division of Urology, Department of Surgery, Harvard Medical School, Boston, Massachusetts, USA.; ^5^ Department of Men's Health Research, Gulf Medical University, Ajman, UAE.

**Keywords:** COVID-19, SARS-CoV-2, coronavirus, deaths, male, testosterone deficiency

## Abstract

Although not universal, many epidemiological data sources signal that a higher proportion of males than females with confirmed Severe Acute Respiratory Syndrome Coronavirus 2 (SARS-CoV-2) infections have adverse outcomes, such as intensive care unit (ICU) admission and death. Though likely multifactorial, the various hypotheses that have been proposed as underlying factors behind this trend are related to greater smoking prevalence among males, testosterone (T) deficiency causing an inflammatory storm, androgen-driven pathogenesis of SARS-CoV-2, a protective effect of estrogen in females, and inborn errors of cytokine immunity. This review aims at examining the evidence and at assessing the likelihood that the factors being investigated are contributory to the reported trend of male predominance of severe COVID-19 cases. Sources were obtained using the PubMed database and were selected based on their relevance to one of the primary hypotheses attempting to explain the strong male sex bias of severe SARS-CoV-2 infections. Emphasis was placed on meta-analyses and population-based studies. Sources are current through February 22, 2022. A severe COVID-19 case or outcome is defined in this review as a progression of the SARS-CoV-2 virus that results in either admission to an ICU for management of symptoms and clinical stabilization or which leads to death. Although the trend of male predominance of severe COVID-19 cases is likely multifactorial, the hypothesis of T deficiency causing an inflammatory storm has support from many studies with limited conflicting evidence. An inborn error in cytokine immunity is also well supported, but it needs more studies to add support to the hypothesis. The immunologic protective effect of estrogen is supported by multiple studies, but it also has conflicting evidence. It appears less likely that the trend is caused solely by an increased prevalence of smoking among males or an androgen-driven pathogenesis, based on the extent of conflicting evidence.

## Introduction

Severe Acute Respiratory Syndrome Coronavirus 2 (SARS-CoV-2) is a highly infectious virus that is the cause of the current pandemic. Once infecting its human host, the virus produces a constellation of symptoms, commonly referred to as COVID-19, which often impacts a great variety of body systems. Symptoms of COVID-19 can include cough, ageusia, anosmia, body aches, fever, chills, fatigue, sore throat, headache, shortness of breath, coagulopathy, and acute hypoxic respiratory failure, among others.^1^

The severity of COVID-19 ranges dramatically from relatively mild cold and flu-like symptoms to the development of acute respiratory distress syndrome (ARDS) and significant organ dysfunction, leading to death. Elderly age and significant co-morbidities such as obesity, pregnancy, history of heart failure or chronic lung disease, diabetes, chronic kidney disease, or cancer are more likely to result in a severe adverse outcome as a result of infection with the SARS-CoV-2 virus.^2^

It is reported that the virus uses the receptor angiotensin-converting enzyme 2 (ACE2) for cell entry and the transmembrane serine protease 2 (TMPRSS2) for S protein priming, together allowing for viral entry into host cells.^3^ However, other docking and receptor proteins are also utilized by the SARS-CoV-2 virus to facilitate host cell entry and propagation of the viral genome.^4^

Many global epidemiological data sources indicate that males account for a higher proportion of severe SARS-CoV-2 infections than females, despite roughly equivalent infection rates (Table 1).^5,6^ Severe COVID-19 cases and outcomes are defined in this review as a progression of the SARS-CoV-2 virus to an extent that it results in either admission to an intensive care unit (ICU) for management of symptoms and clinical stabilization or which leads to death with infection of the SARS-CoV-2 virus as the precipitating factor.

**Table 1.** Evidence of Male Predominance of Severe COVID-19 Outcomes

Authorship	Year	Sample size	Research type	Significant findings
Harvard Gender Lab	2021	41,877,107	Cross-Sectional	COVID-19 mortality rate in the United States is 54.9% male and 45.1% female. COVID-19 case rate in the United States is 48.5% male and 51.5% female.
Jin et al.	2020	1580	Case Series	Independent of age, men with SARS-CoV-2 infection have a greater risk of worse outcomes than females despite nearly equivalent prevalence.
Peckham et al.	2020	3,111,744	Meta-Analysis	Men with SARS-CoV-2 infection have nearly three times the odds of requiring ICU admission and also had a greater risk of death compared with females.
Traish and Morgentaler	2020	Unspecified	Review	Several countries and 23.5% of states in the United States reported a female predominance of severe SARS-CoV-2 infections.

ICU, intensive care unit; SARS-CoV-2, severe acute respiratory syndrome coronavirus 2.

Although this male predominance of severe SARS-CoV-2 infections is not reported universally, it is noted to be highly prevalent among many nations and states who closely track these data and publish their results; therefore, it merits further investigation. As an example, a large meta-analysis of 3.1 million patients with confirmed SARS-CoV-2 infections reported that males had nearly three times the odds (OR = 2.84; 95% confidence interval [CI] = 2.06–3.92) as females of requiring ICU admission and also had higher odds of death (OR = 1.39; 95% CI = 1.31–1.47) despite a roughly equivalent incidence of SARS-CoV-2 infections between males and females.^7^

In the United States, the overall proportion of COVID-19-related deaths has been reported as 54.9% male and 45.1% female as of November 29, 2021 according to the Gender Lab at Harvard School of Public Health, although various states have demonstrated a female predominance.^6^ The Gender Lab reports that during the measured period, males accounted for 48.5% of COVID-19 cases versus 51.5% for females.^6^ Comparable ratios of male predominant COVID-19 deaths have also been observed in many countries globally.^5,7^ Of note, developed countries such as Canada, Portugal, Finland, and Vietnam as well as 23.5% of states within the United States reported a female predominant death toll secondary to COVID-19.^8^ A deeper understanding of the nature of this trend may provide important clues about the pathogenesis of SARS-CoV-2 as well as potential therapeutic drug targets and other effective treatments after infection.

## Methods

This review aims at examining the evidence and assessing the likelihood that the factors being investigated are contributory to the reported trend of male predominance of severe COVID-19 cases secondary to infection with SARS-CoV-2. Evidence for this review was obtained using the PubMed database managed by the United States National Library of Medicine and is current through February 22, 2022. Published manuscripts utilized as the basis of this review include data sets from many countries and are not limited to the United States. Database search criteria included the term “COVID” or “SARS-CoV-2” paired with either “smoking,” “testosterone,” “androgen,” “estrogen,” “male predominance,” or “cytokine immunity.”

Of the 7540 manuscripts meeting such search criteria, 80 were selected for analysis and inclusion based on their relevance to the stated aim of this review. Emphasis was placed on meta-analyses and population studies, when available, so as to utilize the most current and comprehensive data sets. Proportions of males and females with severe outcomes, including death secondary to infection with the SARS-CoV-2 for the United States, were collected using data from the Gender Lab at Harvard School of Public Health and is current through November 13, 2021. All CIs of data incorporated in this review have a 95% probability that the actual value falls within the specified range.

## Parallels to the Past

The reported male sex bias of severe SARS-CoV-2 infections parallels a trend of male predominant mortality secondary to infection by the 2002–2003 Severe Acute Respiratory Syndrome Coronavirus 1 (SARS-CoV-1).^9^ A study of SARS-CoV-1 infected patients indicated that male and female mortality rates were 21.9% and 13.2% (*p* < 0.0001), respectively, with an age-adjusted relative risk of 1.62 (95% CI 1.21–2.16).^9^

The male predominant mortality trend secondary to SARS-CoV-1 infection was successfully replicated in mice by Channappanavar et al.^10^ The authors reported that infecting a middle-aged cohort of both male and female mice with a highly infectious dose of 5000 plaque-forming units of mouse-adapted SARS-CoV-1 resulted in a male and female mortality rate of 90% and 20%, respectively, independent of B and T cell responses.^10^ On analysis of the infected mouse lungs in the middle-aged cohort, the authors noted that the male mice had both a two- to threefold higher inflammatory cell recruitment and a four- to fivefold higher total neutrophil count, relative to that of the infected female mice.^10^

This relative increase in inflammation among SARS-CoV-1 infected male mice could have accounted for the drastically increased mortality rate relative to female mice due to the effects of a potential inflammatory storm. Although human studies have not been performed that directly verify these results, the data of these studies are suggestive that a similar pattern of male predominant mortality exists between patients infected with SARS-CoV-1 and SARS-CoV-2.

## Factors Accounting for Trend of Male Predominant Severe COVID-19 Outcomes

Hereafter, this review will address five factors that have been proposed as potential contributors to the observed male predominance of severe COVID-19 cases secondary to SARS-CoV-2 infection. The factors assessed in this review pertain to either a higher prevalence of tobacco smoking among males, testosterone (T) deficiency-induced inflammatory storm, androgen-driven pathogenesis of severe SARS-CoV-2 infections, the protective immunologic effect of estrogen in females, and also genetic factors including various inborn errors of cytokine immunity that are present in some people.

### Tobacco smoking prevalence

Evidence for this section is based on a review of eight manuscripts, which include a meta-analysis and two large retrospective cohort studies (Table 2). The sample size providing these data is greater than 7,913,491 people.

**Table 2.** Evidence of Tobacco Smoking Accounting for Male Predominance of Severe COVID-19 Outcomes

Authorship	Year	Sample size	Research type	Significant findings
Chakladar et al.	2020	71	Review	ACE2 and TMPRSS2 were increased in current smokers, relative to former smokers.
Channappanavar et al.	2017	Unspecified	Basic Science	Male-predominant mortality of SARS-CoV-1 demonstrated in mice subjects without exposure to cigarette smoke.
Gao et al.	2022	7,869,534	Retrospective Cohort	Current smokers have increased COVID-related complications compared with never-smoker COVID-19 patients.
Ismail et al.	2022	5889	Observational	Current smokers have increased COVID-related complications compared with never-smoker COVID-19 patients.
Leung et al.	2020	449	Basic Science	Smokers and those with COPD have increased airway expression of ACE2.
Patanavinch et al.	2020	11,590	Meta-Analysis	Tobacco smoking is associated with progression of COVID-19.
Wilkinson et al.	2021	25,958	Retrospective Cohort	Former smokers, but not current smokers, had a significantly higher risk of COVID-19-related 30-day mortality.
World Health Organization	2021	Unspecified	Cross-Sectional	Globally, the prevalence of tobacco use is ∼40% in males and 9% in females.

ACE2, angiotensin converting enzyme 2; SARS-CoV-1, severe acute respiratory syndrome coronavirus 1; TMPRSS2, transmembrane serine protease 2.

Tobacco smokers and those with chronic obstructive pulmonary disease have increased airway expression of ACE2 and TMPRSS2, which together are among the mechanisms believed to facilitate the entry and viral genomic incorporation of SARS-CoV-2 into host cells.^11,12^ Further, levels of ACE2 and TMPRSS2 have been reported in higher concentrations of active smokers relative to former smokers.^12^ A recent meta-analysis of 11,590 patients with confirmed SARS-CoV-2 infection indicates that smoking is associated with a progression of the virus (OR 1.91; 95% CI 1.42–2.59), thereby leading to more severe outcomes.^13^

However, a recent large study of 25,968 American Veterans hospitalized with confirmed SARS-CoV-2 infection reported that there was no statistical difference in 30-day mortality between subjects who reported that they were currently smoking relative to those who reported that they had never smoked, although findings were reported to be significant in those who reported that they were previous, but not current, smokers.^14^ Another recent study of 5889 COVID-19 patients identified that, compared with those who had never smoked, current tobacco smoking increases risk of ARDS (OR 1.69, 95% CI 1.09–2.55), renal injury (OR 1.55, 95% CI 1.10–2.14), and acute liver injury (OR 1.33, 95% CI 1.01–1.74), but that the increased risk of developing these conditions did not significantly impact disease outcome.^15^

Of note, a recent cohort study including nearly 7.9 million people in the United Kingdom reported that compared with those who had never smoked, current smokers had a lower risk of COVID-related ICU admission (HR 0.31, 95% CI 0.24–0.40) and death (HR 0.79, 95% CI 0.70–0.89).^16^ This UK study further reported that former smokers had a slightly higher risk of COVID-related ICU admission (HR 1.17, 95% CI 1.04–1.31) and death (HR 1.17, 95% CI 1.10–1.24) than those who had never smoked, but that all-cause mortality was higher for current smokers than former smokers (HR 1.42, 95% CI 1.36–1.48).^16^

This suggests that current tobacco use may convey some form of protective effect against developing a severe case of COVID-19, but that smoking still increases the likelihood of mortality secondary to the negative impact on health related to tobacco use. A variety of mechanisms by which current tobacco use may protect against severe SARS-CoV-2 infection have been proposed and include a potential nicotinic downregulation of the ACE2 receptor, anti-inflammatory properties of nicotine, and nicotine's reported reduction in cytotoxic T-lymphocytes.^16^ More data from population-based studies are needed to confirm these findings.

According to the World Health Organization, ∼40% of men globally smoke as compared with nearly 9% of women.^17^ Therefore, the male predominance of severe COVID-19 cases could be partially explained by the vastly greater proportion of males in the global population who smoke tobacco products relative to females. However, if history of tobacco use is the sole cause of this trend it would be expected that the COVID-19 male mortality rate would be much greater and thus more reflective of the fourfold greater population of male smokers than female smokers.

Moreover, Channappanavar et al^10^ demonstrated male predominant mortality in mice infected with SARS-CoV-1, which parallels the observed trend of severe SARS-CoV-2 infection, despite the mice having no exposure to cigarette smoke. These findings suggest that broader pathophysiological mechanisms beyond a history of tobacco use may be involved in the noted trend of male predominance of severe COVID-19 cases. However, history of tobacco use, particularly current tobacco use, may still be a contributing factor in the prognosis.

### T deficiency

Evidence for this section is based on a review of 26 manuscripts, which include a randomized-controlled trial and many prospective and retrospective cohort studies (Table 3). The sample size providing these data is greater than 4331 people. Although specific criteria may vary by study, low T is typically defined as clinical hypogonadism when it reaches a level below 300 ng/dL.^18^

**Table 3.** Evidence of Testosterone Deficiency Accounting for Male Predominance of Severe COVID-19 Outcomes

Authorship	Year	Sample size	Research type	Significant findings
Beltrame et al.	2022	120	Retrospective Cohort	COVID-19 males with low T are more likely to develop ARDS with associated in-hospital mortality than those with normal T.
Bobjer et al.	2013	40	Nested Cross-Sectional	Low T is associated with increased TNF-α and other inflammatory markers.
Cinislioglu et al.	2022	358	Prospective Cohort	Serum total T was significantly lower in severe COVID-19 cohorts relative to mild- and moderate-COVID-19 cohorts.
Di Stasi et al.	2022	17	Retrospective Cohort	Higher T levels in women are correlated with a more robust inflammatory response.
Fernandez et al.	2019	Unspecified	Review	Obesity is a significant risk factor for T deficiency due to the suppression of the hypogonadotropic axis.
Garawi et al.	2014	Unspecified	Cross-Sectional	Globally, obesity is more prevalent among females than males.
Heffernan et al.	2011	648	Prospective Cohort	Low T and high estradiol increases inflammation and is associated with ARDS in males and females.
Iglesias et al.	2014	150	Observational	About half of men admitted for acute illness had low T, which subsequently significantly increased risk of death.
Iglesias et al.	2015	150	Prospective Cohort	Hypogonadism is a strong independent predictor of both all-cause and cardiovascular mortality in elderly men.
Isidori et al.	1999	38	Prospective Cohort	T levels were 30–40% lower in obese males compared with controls.
Kelly et al.	2013	Unspecified	Basic Science	Testicular-feminized mice had increased levels of TNF-α and IL-6 relative to non-feminized littermates.
Ma et al.	2021	404	Observational	Males with COVID-19 had low T and significantly decreased T:LH ratio, leading to increased inflammatory markers.
Maggio et al.	2014	108	Randomized Controlled Trial	Transdermal T treatment is not associated with significant changes in inflammatory markers.
Malkin et al.	2004	27	Clinical Trial	T caused reductions in TNF-α and IL-1β and also induced an increase in IL-10.
Mohan et al.	2015	1768	Observational	Both T and dihydrotestosterone were independently associated with higher forced expiratory volume in 1 sec and forced vital capacity.
Pan et al.	2020	34	Observational	COVID-19 was not detected in semen samples of infected males 30 days after confirming infection.
Papadopoulos et al.	2021	Unspecified	Review	Half of men aged 65 and older hospitalized for acute respiratory illness were found to have low T.
Pozzilli and Lenzi	2020	Unspecified	Review	T reduces pro-inflammatory cytokines.
Mohamad et al.	2019	Unspecified	Review	Hypogonadism is associated with increased pro-inflammatory cytokines.
Rastrelli et al.	2021	31	Prospective Cohort	Low T is predictive of worse outcomes, leading to increased likelihood of ICU transfer or death.
Salonia et al.	2022	121	Retrospective Cohort	More than 50% of men had clinical hypogonadism 7 months after recovery from SARS-CoV-2 infection.
Schroeder et al.	2020	35	Retrospective Cohort	68.6% of COVID-positive males had low T on hospital admission.
Siiteri	1987	Unspecified	Review	Androstenedione is efficiently converted to esterone in adipose tissue.
Traish and Morgentaler	2020	Unspecified	Review	T suppresses inflammatory cytokines such as IL-6.
Vena et al.	2021	221	Observational	Low T is correlated with greater inflammatory markers and greater likelihood of COVID-related respiratory distress.
Zheng et al.	2022	61	Basic Science	Low T, particularly in the second week after COVID-19 symptom onset, is predictive of COVID-19 severity.

ARDS, acute respiratory distress syndrome; IL, interleukin; LH, luteinizing hormone; T, testosterone; TNF, tumor necrosis factor.

Pro-inflammatory cytokines, such as interleukin (IL)-6, play a prominent role in the progression of COVID-19 and have been implicated in inducing cytokine storm related to severe respiratory illness.^8^ As T has been shown to suppress the expression of such pro-inflammatory cytokines, a T deficiency could, therefore, increase patient vulnerability to severe COVID-19 outcomes by mitigating the effectiveness of one of the body's natural protective mechanisms against cytokine storm.^8,19^ This hypothesis is supported by several animal and human subject studies that report that hypogonadism is associated with increased pro-inflammatory cytokines and that treatment with T reduces IL-1-beta, IL-6, C-reactive protein (CRP), and tumor necrosis factor-alpha (TNF-α).^20–24^ However, the magnitude and clinical impact of T's anti-inflammatory properties remains debatable.^20^

Contrary to the effect seen in men, one small study reports that higher T levels in women with COVID-19 pneumonia have been reported to be correlated with a more robust inflammatory response.^25^

Multiple studies have demonstrated that low T is predictive of worse outcomes in COVID-19 patients, leading to an increased likelihood of ICU transfer or death.^8,26–31^ A similar trend was observed in the previously discussed study using mice infected with SARS-CoV-1, which reported that many of the male mice had low T, a finding correlating with significant male predominance of post-infection fatalities in the study.^10^ Interestingly, the hypogonadal state noted in patients with SARS-CoV-2 infection has been reported to endure long after recovery from the virus.^28,32^

Patients with confirmed SARS-CoV-2 infection also have significantly elevated luteinizing hormone (LH) and a significantly decreased T:LH ratio, suggesting that SARS-CoV-2 infection may lead to testes dysfunction in the form of damage to Leydig cells.^33,34^ This low T:LH ratio is correlated with increased inflammation, because this ratio is negatively associated with the quantity of white blood cells and CRP levels.^33^ Although multiple studies have reported that SARS-CoV-2 was not detected in the semen of males with confirmed infection, it cannot be definitively determined that the testes are not a target of the virus because the endocrine functions of the testes are independent of its exocrine functions.^33,35^

It is unclear whether SARS-CoV-2 infection induces a hypogonadal state or whether T deficiency predisposes males to severe COVID-19 outcomes. This determination is made more difficult, as there are limited studies that report T levels in subjects both pre- and post-infection with SARS-CoV-2.

Higher T and dihydrotestosterone levels are also independently associated with higher forced expiratory volume in 1 sec and forced vital capacity in middle-aged community dwelling men without SARS-CoV-2 infection.^36^ Assuming these data also apply to males with SARS-CoV-2, it can be inferred that men with higher T levels may have improved lung function and, consequently, better COVID-19 outcomes relative to hypogonadal men.

Along with advanced age and other factors, obesity is a significant risk factor for T deficiency in males.^37^ This is due to obesity-induced increases in levels of leptin, insulin, proinflammatory cytokines, and estrogen, which can cause a functional hypogonadotropic hypogonadism resulting in disruption of the hypothalamic gonadotrophin-releasing hormone neurons.^37,38^ Obesity has been shown to decrease T levels as much as 40% compared with controls.^38^

Although obesity is also connected with other comorbidities, which could result in more severe COVID-19 outcomes, it is likely not the sole cause of this male sex bias of severe cases as the average prevalence of obesity globally is 10% in males and 18% in females.^39^ In addition, female prevalence of obesity was found to exceed that of males in 87% of the 151 countries studied.^39^ If obesity were the sole predominant factor in determining the severity of COVID-19 outcomes, it would be expected that the observed sex bias would be reversed, presenting as a female predominance rather than a male predominance. Further, if the observed sex bias were attributed solely to a protective effect of estrogen, as will be discussed hereafter, it would be expected that obesity would be somewhat of a protective factor against severe SARS-CoV-2 infections. This is due to the efficient conversion of androstenedione to estrone in adipose tissue, which would increase the total amount of estrogen in an obese individual.^40^

A pro-inflammatory sex hormone profile of low T and high estradiol has been reported to be associated with ARDS in both males and females in a non-COVID population, although this failed to impact mortality rate.^41^ Of note, hypogonadism is reported to be a strong independent predictor of all-cause mortality (HR 3.35; 95% CI 1.55–7.23; *p* = 0.002).^42^ Multiple studies indicate that a low T hypogonadal state leads to worse outcomes in patients with acute respiratory illnesses.^41–43^ Half of the males older than 65 years of age admitted to the hospital for acute illnesses such as a respiratory tract infection have been reported to have low T.^43,44^

Thus, the data in support of a T deficiency leading to worse outcomes in men infected with SARS-CoV-2 or other acute respiratory illnesses are quite strong, with limited conflicting evidence. However, having low T levels alone may not provide a complete explanation of the observed male predominance of severe COVID-19 cases because females at baseline typically have lower levels of T than males, which would thereby logically favor a female predominance to severe infections. More data are needed to identify the specific mechanism by which a hypogonadal state in males leads to worse outcomes in patients infected with SARS-CoV-2.

### Androgen-driven pathogenesis of SARS-CoV-2

Evidence for this section is based on a review of 18 manuscripts, which include two cross-sectional studies and many observational studies (Table 4). The sample size providing these data is greater than 9466 people.

**Table 4.** Evidence of an Androgen-Driven Pathogenesis of Severe Acute Respiratory Syndrome Coronavirus 2 Accounting for Male Predominance of Severe COVID-19 Outcomes

Authorship	Year	Sample size	Research type	Significant findings
Aguiar et al.	2020	515	Basic Science	ACE2 is not prominent in epithelial lung tissue. Receptors other than ACE2 and TMPRSS2 are involved in SARS-CoV-2 host cell entry.
Baratchian et al.	2020	Unspecified	Review	There is no difference in TMPRSS2 expression in lung epithelial cells between males and females.
Baghani et al.	2021	164	Cross-Sectional	The severity of AGA did not correlate with the severity of COVID-19 among hospitalized patients.
Bhowmick et al.	2020	Unspecified	Review	TMPRSS2 is an androgen-responsive receptor that is highly prevalent in the male prostate tissue.
Fan et al.	2020	Unspecified	Basic Science	ACE2 is highly expressed in renal tubular cells, Sertoli cells, Leydig cells, and cells of testicular seminiferous ducts.
Gedeborg et al.	2021	474	Case-Control	The increased mortality from COVID-19 in men with PCa treated with ADT is due to confounding factors.
Ghafoor et al.	2021	300	Prospective Cohort	The AGA severity was significantly correlated with COVID-19 severity in men as compared with women.
Goren et al.	2020	41	Observational	71% of patients with bilateral COVID-19 pneumonia showed signs of AGA.
Jiménez-Alcaide et al.	2021	2280	Retrospective Cohort	ADT in male PCa patients was not significantly correlated with severity of COVID-19 outcome.
Kim et al.	2011	131	Comparative	African Americans have higher rates of aggressive PCa than Caucasians.
Lucas et al.	2014	32	Review	TMPRSS2 is expressed abundantly in high-grade PCas and PCa metastases.
McCoy et al.	2020	Unspecified	Comparative	African Americans have higher rates of AGA than Caucasians.
Mjaess et al.	2020	Unspecified	Review	ACE2 and TMPRSS2 are some of the primary receptors by which the SARS-CoV-2 virus attains host cell entry.
Montopoli et al.	2020	4532	Retrospective Cohort	PCa patients receiving ADT had a significantly reduced risk of COVID-19 infection.
Pan et al.	2020	34	Observational	Only sparse expression of ACE2 and TMPRSS2 was noted in testes of COVID-19 infected males.
Thebault et al.	2020	963	Cross-Sectional	African-American communities have much higher COVID-19 infection and death rates than other ethnicities.
Traish and Morgentaler	2020	Unspecified	Review	TMPRSS2 expression is not found to be increased in human epithelial lung tissues.
Wambier and Goren	2020	Unspecified	Review	Androgen expression is low in pre-pubertal children.

ADT, androgen deprivation therapy; AGA, androgenetic alopecia; PCa, prostate cancer.

It has been reported and thoroughly investigated that SARS-CoV-2 utilizes a two-part mechanism for host cell entry and genomic incorporation into host cells utilizing ACE2 and TMPRSS2, although it is likely that other mechanisms of viral cell entry are also involved.^4,45^ Some studies have reported that TMPRSS2 is an androgen-regulated cell-surface serine protease that is highly expressed in lung and prostate epithelial tissues.^46,47^ However, multiple subsequent human and murine studies have reported no increased expression of TMPRSS2 in lung tissues and also reported no statistical difference in TMPRSS2 expression between men and women.^4,8,44,48–50^ These more recent studies collectively suggest that TMPRSS2 expression is not a prominent factor contributing to the male predominance of severe COVID-19 cases, as the lungs are typically highly impacted by the SARS-CoV-2 virus.

The U.S. counties with majority African-American populations have been reported to have a threefold higher SARS-CoV-2 infection rate and nearly a fourfold higher COVID-19 mortality rate relative to counties with predominantly Caucasian populations.^51^ Deaths per hundred thousand people for counties consisting of a majority Caucasian, Asian, African American, and Latino populations were 1.1, 0.4, 6.3, and 0.6, respectively.^51,52^

Although the causes of these increased infection and mortality rates among African American populations are likely multifactorial, including socioeconomic factors, it is worth noting that other androgen-driven pathologies such as aggressive prostate cancer (PCa) and androgenetic alopecia (AGA) also tend to impact African American males in a similar distribution.^52,53^ Although there is potentially an androgenic component to SARS-CoV-2 infection, the lack of abundant expression of TMPRSS2 in the lung epithelium as noted earlier makes it less likely that this reported ethnic disparity of severe COVID-19 outcomes is attributed to an androgenic cause.

If the pathogenesis of severe SARS-CoV-2 infection is highly influenced by androgens, the low degree of androgen receptor expression before puberty may partially explain the relatively low incidence of severe COVID-19 outcomes among pre-pubertal children.^54,55^ However, although several studies have reported that new hair loss believed to be consistent with AGA has been observed in hospitalized COVID-19 patients, it is more likely, given the extensive reports of COVID-related T deficiency, that the alopecia was not driven by an androgenic cause.^56,57^ This argument is supported by a more recent study that reported that the severity of AGA is not correlated with the severity of COVID-19 outcomes among hospitalized patients.^58^

A single study reported that male COVID-19 patients with PCa treated with androgen deprivation therapy (ADT) had both a lower incidence of SARS-CoV-2 infections and lower rates of severe COVID-19 outcomes, such as death, relative to PCa patients who did not receive ADT.^59^ However, these data largely stand in isolation as more recent studies of mixed sample sizes report data that refute this finding.^60,61^ Further, the results from the single study are potentially confounded by the likelihood that men with known PCa managed with ADT may have less energy at baseline or may have taken greater precautions to social distance and follow recommended health guidelines, as compared with the general population.

Although there may be some degree of an androgen-driven component of the SARS-CoV-2 mechanism facilitating viral host cell entry and viral genomic incorporation into the host, these data are relatively weak and signal that this mechanism likely is not a significant factor in determining the male predominance of severe SARS-CoV-2 infections.

### Estrogen immunologic protective effect

Evidence for this section is based on a review of 18 manuscripts, which include two large meta-analyses and several cohort studies (Table 5). The sample size providing these data is greater than 475,723 people.

**Table 5.** Evidence of Immunologic Protective Effect of Estrogen Accounting for Male Predominance of Severe COVID-19 Outcomes

Authorship	Year	Sample size	Research type	Significant findings
Baristaite and Gurwitz	2022	Unspecified	Basic Science	17-β-estradiol may reduce SARS-CoV-2 infection of lung epithelial cells.
Beltrame et al.	2022	120	Retrospective Cohort	Increased estrogen levels were positively associated with risk of COVID-related death in both males and females.
Breithaupt-Faloppa et al.	2020	Unspecified	Review	Estrogen increases number of T-reg cell populations.
Cabrera et al.	2021	5440	Review	Females have a higher incidence of long-COVID syndrome than males.
Channappanavar et al.	2017	Unspecified	Basic Science	Gonadectomy or tamoxifen treatment significantly increased mortality rate of SARS-CoV-1 infected female mice.
Di Stasi et al.	2022	17	Observational	COVID-19 females with higher T levels were more likely to demonstrate a profound inflammatory response.
Grandi et al.	2020	Unspecified	Review	Estrogens stimulate the humoral response to viral infections.
Khan et al.	2015	Unspecified	Review	Estrogen receptors are highly expressed on T and B cells.
Kovats et al.	2012	Unspecified	Review	Estradiol promotes dendritic cell differentiation during periods of inflammation.
Lahm et al.	2008	Unspecified	Review	Estrogen causes vasodilation of pulmonary vasculature and attenuates the hypoxia vasoconstrictor response.
Lassi et al.	2021	31,016	Meta-analysis	SARS-CoV-2 virus yields poorer maternal and fetal outcomes, relative to pregnant mothers not infected with the virus.
Ortona et al.	2021	Unspecified	Review	Women younger than the age of 60 are twice as likely to develop long-COVID syndrome than men.
Ruggieri et al.	2020	Unspecified	Review	Estrogens stimulate humoral response to viruses by inducing higher levels of antibody production.
Scotland et al.	2011	Unspecified	Basic Science	Estrogen enhances macrophage recruitment, but it does not significantly impact T-lymphocyte populations.
Stanczyk et al.	2019	24	Observational	Pre-menopausal women have higher estrogen levels than post-menopausal women.
Sudre et al.	2021	558	Review	Higher body mass index and female sex are independent risk factors of long-COVID syndrome.
Wang et al.	2021	Unspecified	Review	Pregnant women with SARS-CoV-2 are more likely to have a complicated pregnancy than pregnant women without COVID-19.
Wei et al.	2021	438,548	Meta-Analysis	COVID-19 significantly increases maternal and neonatal complications compared with pregnant females without COVID-19.

An alternative hypothesis for the observed pattern of male predominance of severe COVID-19 outcomes is that females are more protected against such negative outcomes due to hormonal factors. It has been proposed that estradiol and synthetic estrogens, such as ethinylestradiol, may convey a protective benefit in women against severe COVID-19 outcomes.^62^ This protective benefit is believed to be particularly beneficial among pre-menopausal women who have higher estrogen levels.^62^

In addition to estrogen being one of the primary hormones involved in female sexual development and maturation, estrogen receptors are also highly expressed on T and B cells.^63^ Estrogens stimulate the humoral response to viral infections by both inducing higher levels of antibodies and activating antibody-producing cells.^64^ However, a recent observational study indicates that higher estradiol levels are associated with a higher probability of COVID-related mortality in both males and females.^30^

It has been demonstrated that leukocyte activity in females, relative to males, more rapidly detects and eliminates pathogens, recruits greater numbers of macrophages and toll-like receptors, is more efficient at nicotinamide adenine dinucleotide phosphate oxidase killing, and also has increased populations of resident anti-inflammatory T-lymphocytes.^65–67^ In addition, estradiol signals the production of new dendritic cells during inflammation that initiate all the antigen-specific immune responses against invading pathogens.^68–70^ The more efficient leukocyte function and greater influence on macrophages in females has been proposed to allow aggressive recognition and elimination of diverse infectious stimuli without recruitment of circulating neutrophils or excessive cytokine production.^65^

However, multiple studies have reported that females have a higher incidence than males of Long-COVID Syndrome, loosely defined as COVID-19 symptoms persisting for greater than 4 weeks, although specific definitions vary between studies.^71,72^ This, therefore, suggests that the hormonal protective factors of females may be less effective at eliminating the SARS-CoV-2 virus.

Aside from the protective benefit of estrogen on the immune system, estrogen causes vasodilation in the pulmonary vasculature and attenuates the vasoconstrictor response to various stimuli, including hypoxia.^73^ Moreover, a study has reported that estradiol may decrease SARS-CoV-2 infection severity in lung epithelial cells by decreasing the expression of ACE2 and TMPRSS2.^74^ As COVID-19 can lead to critical respiratory failure, estrogen may also play an important role in partially mitigating the severity of the disease by maintaining adequate blood flow in the lungs for gas exchange, thereby minimizing hypoxia.

To demonstrate the relationship between SARS-CoV-1 and gonadotropic hormones, Channappanavar et al. infected middle-aged male and female mice with flutamide and tamoxifen, respectively, or performed a gonadectomy in both groups.^10^ Significantly reducing the quantities of sex hormones in this manner demonstrated no significant effect on male mortality but significantly increased the female mortality rate to 85%, relative to 20% in the control group at the same time point.^10^ The authors interpreted this as signifying that androgens likely do not play a significant role in the pathological progression of SARS-CoV-1 virus while claiming that the estrogen receptor exhibits a protective effect against SARS-CoV-1 infection.^10^

In addition, the authors noted that their experiments demonstrate that SARS-CoV-1 infection significantly reduces T levels in the control groups, although the data were not included in the published study to quantify this decrease.^10^ Although comparable studies have yet to be performed using SARS-CoV-2 as the pathogen, there are many known parallels between the mechanism of the two coronaviruses and there is a high likelihood that estrogen may play a similar role in protecting against severe COVID-19 outcomes.

However, unlike T deficiency, which has been reported in some series to increase risk of severe adverse outcomes including death as a result of SARS-CoV-2 infection, there is no current evidence in human studies that quantifiably demonstrates that an estrogen deficiency in premenopausal women leads to comparable rates of adverse outcomes related to COVID-19.

The potential immunologic protective benefit of estrogen as the sole cause of the observed male predominance of severe COVID-19 cases is challenged by the fact that SARS-COV-2 infection in pregnant females has been reported to have a significantly increased risk of both maternal and fetal complications.^75–77^ A large meta-analysis incorporating data from 438,538 pregnant women reported that maternal SARS-CoV-2 infection is associated with preeclampsia (OR 1.33, 95% CI 1.03–1.73), preterm birth (OR 1.82, 95% CI 1.38–2.39), and stillbirth (OR 2.11, 95% CI 1.14–3.90).^75^

The degree of complications in this cohort was found to be directly correlated with the severity of the SARS-CoV-2 infection in the mother.^75^ A subsequent meta-analysis of 31,016 pregnant women reported similar findings, which demonstrated that the SARS-CoV-2 virus yields poorer maternal and fetal outcomes, relative to pregnant mothers not infected with the virus.^76^ As estrogen levels in females are known to dramatically increase during pregnancy, it would be expected, according to the immunologic protective effect of estrogen hypothesis, that pregnant females would experience a relative sparing of severe SARS-CoV-2 infections. However, this logical assumption is clearly refuted by the data.

Thus, although there are significant data supporting the immunologic protective effect of estrogen in women, data are mixed about whether this effect results in improved outcomes of women infected with SARS-CoV-2 relative to men who have baseline lower estrogen concentrations.

### Inborn errors of cytokine immunity

Evidence for this section is based on a review of three manuscripts, which include several case-control studies as well as a bench research study (Table 6). The sample size providing these data is 4120 people.

**Table 6.** Evidence of Inborn Errors of Cytokine Immunity Accounting for Male Predominance of Severe COVID-19 Outcomes

Authorship	Year	Sample size	Research type	Significant findings
Bastard et al.	2020	2877	Case Control	In patients with severe SARS-CoV-2 infection, 12.5% of males compared with 2.6% of females had inborn errors of type I interferon immunity. These autoimmune antibodies were practically absent from the cohorts with mild SARS-CoV-2 infection or without such infection.
Hadjadj et al.	2020	50	Case Control	Patients with severe SARS-CoV-2 infection were found to have significantly impaired type I interferon responses, resulting in increased serum SARS-CoV-2 viral load and also an increased inflammatory response.
Zhang et al.	2020	1193	Bench	An enrichment in loss of function mutations in the interferon response is associated with severe SARS-CoV-2 infection.

As none of the models discussed earlier decisively are the sole cause accounting for this trend of male predominance of severe SARS-CoV-2 outcomes, it is possible that some individuals and families have a genetic predisposition to greater systemic inflammatory processes after infection with SARS-CoV-2. A study of 987 males and females with severe COVID-19 pneumonia reported that 12.5% of males relative to 2.6% of females had a B cell autoimmune antibody (auto-Abs) that hindered the individual's ability to block SARS-CoV-2 infection *in vitro* by neutralizing high concentrations of type I interferons.^78^

Notably, these auto-Abs were not identified in any of the 663 individuals with confirmed SARS-CoV-2 infection who were clinically asymptomatic or presented with mild symptoms and were found in only 4 of 1227 healthy individuals not infected with SARS-CoV-2.^78^ These data suggest that a greater proportion of men than women have auto-Abs, which interfere with the Type I interferon response and therefore may account in part for the observed male predominance of severe SARS-CoV-2 infection.

Other small studies reported that patients with severe SARS-CoV-2 infections had significantly impaired Type I interferon responses characterized by no Interferon-β and low Interferon-α production and activity, relative to those with mild or no symptoms after SARS-CoV-2 infection.^79,80^ The effect of this impaired immunity was a persistently increased viral load in the blood and an exacerbated inflammatory response, primarily driven by TNF-α and IL-6.^79^

Although the hypothesis of an inborn error of cytokine immunity may partially account for this trend of male predominance of severe SARS-CoV-2 infections, the sample sizes and number of supporting studies are relatively small. Thus, more investigation is required to determine whether similar findings are found in larger studies and meta-analyses.

## Conclusion

Although not universal, many epidemiological data sources have demonstrated that SARS-CoV-2 infection impacts males more severely than females, yielding a much larger proportion of COVID-19-associated deaths and ICU admissions, despite roughly equivalent infection rates. Various hypotheses have been proposed that strive to uncover the pathophysiological mechanisms accounting for this trend. The extent of conflicting evidence is suggestive that the trend of male predominance of severe COVID-19 outcomes extends beyond solely an elevated proportion of male tobacco smokers relative to female smokers.

The data in support of an androgen-driven pathogenesis of SARS-CoV-2 leading to severe COVID-19 outcomes is also relatively weak. Multiple studies support the trend being attributed to an immunologic protective effect of estrogen in females, although conflicting data are also present. Many studies strongly support, with limited conflicting evidence, that the trend is caused by a T deficiency-induced inflammatory storm.

The hypothesis of an inborn error of cytokine immunity that impairs the Type I interferon response is also well supported, but it requires more investigation to determine whether these findings are supported by studies with larger samples as well as meta-analyses. However, it is possible that this trend is multifactorial with overlapping features of the various hypotheses.

The strengths of this review include robust sample sizes up to 7.9 million patients with confirmed SARS-CoV-2 infection and the incorporation of relevant published meta-analyses and population-based studies. This review is limited by the chronological addition of new variants of SARS-CoV-2 that may operate under varied pathophysiological mechanisms from the original SARS-CoV-2 virus, which was recognized as the source of the present pandemic. In addition, most studies do not report the proportion of each coronavirus variant in their studies.

Reporting of SARS-CoV-2 cases and deaths is not completely standardized across varying states and nations and does not always document the data by gender, which may have altered the overall male and female prevalence of severe COVID-19 outcomes. Vaccination status for SARS-CoV-2 is also typically not reported in manuscripts and is likely a factor that can drastically impact the severity of infection. Despite its limitations, the present work is among the most comprehensive on the topic as it examines the evidence from the most prominent hypotheses accounting for the male predominance of severe COVID-19 outcomes, opposed to solely addressing one hypothesis.

Despite the abundant recent publications related to SARS-CoV-2 and COVID-19 since the start of the pandemic, further investigation is merited to identify how to best continue to reduce the mortality rate of the disease. Additional animal and human studies of subjects infected with SARS-CoV-2 are needed, particularly those with an emphasis on testing the hypotheses presented in this review. Developing a firmer understanding of the pathophysiological mechanisms of SARS-CoV-2 will likely yield important data, which may be used to more specifically target the virus and mitigate the risk of developing severe outcomes secondary to SARS-CoV-2 infection.

## Abbreviations Used

ACE2: angiotensin-converting enzyme 2
ADT: androgen deprivation therapy
AGA: androgenetic alopecia
ARDS: acute respiratory distress syndrome
auto-Abs: autoimmune antibody
CI: confidence interval
CRP: C-reactive protein
ICU: intensive care unit
IL: interleukin
LH: luteinizing hormone
PCa: prostate cancer
SARS-CoV-1: Severe Acute Respiratory Syndrome Coronavirus 1
SARS-CoV-2: Severe Acute Respiratory Syndrome Coronavirus 2
T: testosterone
TNF: tumor necrosis factor
